# Protoporphyrin IX-Dependent Antiviral Effects of 5-Aminolevulinic Acid against Feline Coronavirus Type II

**DOI:** 10.3390/v16101595

**Published:** 2024-10-11

**Authors:** Tomoyoshi Doki, Junna Shimada, Misa Tokunaga, Kaito To, Koichi Orino, Tomomi Takano

**Affiliations:** 1 Laboratory of Veterinary Infectious Disease, School of Veterinary Medicine, Kitasato University, Towada 034-8628, Aomori, Japan; doki@vmas.kitasato-u.ac.jp (T.D.);; 2 Laboratory of Veterinary Biochemistry, School of Veterinary Medicine, Kitasato University, Towada 034-8628, Aomori, Japan; orino@vmas.kitasato-u.ac.jp

**Keywords:** feline coronavirus, 5-aminolevulinic acid, protoporphyrin IX

## Abstract

5-Aminolevulinic acid (5-ALA), a non-proteinogenic amino acid, is an intermediate in the biosynthesis of heme and exerts antiviral effects against feline coronavirus (FCoV); however, the underlying mechanisms remain unclear. In the biosynthesis of heme, 5-ALA is condensed and converted to protoporphyrin IX (PpIX), which is then transformed into heme by the insertion of ferrous iron. Previous research has suggested that the metabolites generated during heme biosynthesis contribute to the antiviral effects of 5-ALA. Therefore, the present study investigated the in vitro mechanisms responsible for the antiviral effects of 5-ALA. The results obtained revealed that 5-ALA and PpIX both effectively reduced the viral titer in the supernatant of FCoV-infected fcwf-4 cells. Moreover, PpIX exerted virucidal effects against FCoV. We also confirmed that 5-ALA increased PpIX levels in cells. While hemin induced heme oxygenase-1 gene expression, it did not reduce the viral titer in the supernatant. Sodium ferrous citrate decreased PpIX levels and suppressed the antiviral effects of 5-ALA. Collectively, these results suggest that the antiviral effects of 5-ALA against FCoV are dependent on PpIX.

## 1. Introduction

Feline coronavirus (FCoV) is an enveloped positive-sense single-stranded RNA virus that belongs to the order Nidovirales, family Coronaviridae, subfamily *Orthocoronavirinae*, genus *Alphacoronavirus* and species *Alphacoronavirus suis* [1]. It has four structural proteins: a spike (S) protein, envelope (E) protein, membrane (M) protein, and nucleocapsid (N) protein [2]. The S protein, which is present on the envelope, is involved in binding to virus receptors and membrane fusion between the cell membrane and envelope. FCoV is classified into two serotypes, types I and II, based on antigenic differences between the the S proteins [3,4,5,6].

FCoV is spreading among domestic cats worldwide. Most FCoV infections are intestinal and rarely fatal. FCoV-infected cats show no clinical signs or develop mild enteritis [7,8,9]. However, FCoV may cause the fatal disease feline infectious peritonitis (FIP) [10,11]. Virulent FCoV, which causes FIP, is considered to emerge due to mutations that increase virulence in the FCoV genome. Although the FCoV genome mutation causing this switch in the pathotype has not been identified, the involvement of an S gene mutation has been suggested [12,13,14,15].

There is currently no vaccine to prevent the onset of FIP. The only treatment that has been effective in cats with FIP is the administration of antiviral drugs. An RNA-dependent RNA polymerase inhibitor (nucleoside analogues) and 3C-like protease inhibitor (GC376) are currently used to treat FIP [16,17,18,19,20,21]. Compounds that exert antiviral effects against FCoV other than these drugs are being investigated for FIP. We recently reported that 5-aminolevulinic acid (5-ALA) inhibited the proliferation of FCoV [22].

5-ALA is an intermediate metabolite in the biosynthetic pathway of heme, a non-proteinogenic amino acid [23]. In animal cells, 5-ALA is synthesized in mitochondria [24]. A tetrapyrrole synthesized from 5-ALA, uroporphyrinogen III, is converted to protoporphyrinogen IX and then to protoporphyrin IX (PpIX), which is subsequently converted to heme by ferrochelatase, which adds Fe^2+^ to PpIX, in mitochondria. Intracellular heme is degraded to biliverdin, CO, and Fe^2+^ by heme oxygenase-1 (HO-1).

A metabolite of 5-ALA may be involved in the antiviral effects of 5-ALA. In cells, 5-ALA induces PpIX. PpIX exerts virucidal effects against enveloped viruses. Human immunodeficiency virus and classical swine fever virus (CSFV) were previously shown to be inactivated by PpIX induced by 5-ALA [25,26]. However, it remains unclear whether 5-ALA exerts antiviral effects against coronavirus, including FCoV, via a similar mechanism [22,27]. Therefore, the present study investigated factors that induce the antiviral effects of 5-ALA against FCoV in vitro using compounds involved in the heme biosynthetic pathway.

## 2. Materials and Methods

### 2.1. Cell Cultures and Viruses

Felis catus whole feus 4 (fcwf-4) cells were kindly supplied by Dr. M. C. Horzinek of the State University of Utrecht, the Netherlands, and were grown in Eagle’s minimum essential medium containing 50% L-15 medium, 100 U of penicillin per mL, and 100 μg of streptomycin per mL. Fetal bovine serum was incorporated into the medium at a concentration of 5% to serve as growth medium and at a concentration of 2% to function as maintenance medium (MM). Type II FCoV strain 79–1146 was supplied by Dr. M. C. Horzinek of the State University of Utrecht, the Netherlands. The virus was grown in fcwf-4 cells at 37 °C.

### 2.2. Compounds

5-ALA and sodium ferrous citrate (SFC) were obtained from Neopharma Japan (Tokyo, Japan). 5-ALA was dissolved at 200 mM and SFC at 50 mM in water. Protoporphyrin IX disodium salt (PpIX) was obtained from Sigma-Aldrich (St. Louis, MO, USA). PpIX was dissolved in dimethyl sulfoxide (DMSO) and adjusted to a concentration of 2.5 mM. Hemin was used as an inducer of HO-1 and was obtained from Tokyo Chemical Industry Co., Ltd. (Tokyo, Japan). It was dissolved in water and adjusted to a concentration of 5 mM.

### 2.3. Cytotoxicity Assay

fcwf-4 cells were seeded at a density of 2 × 10^5^ cells in 100 μL of MM in a 96-well plate. Cells were cultured at 37 °C for 24 h, and the supernatants were then removed. The MM-containing compounds were added to the cells, which were then incubated for 24 h and subsequently washed with MM. Water-soluble tetrazolium 8 (WST-8, Kishida Chemical Co., Ltd., Osaka, Japan), diluted 10-fold with MM, was added to cells at a volume of 100 µL per well. Cells were then incubated at 37 °C for 1 h, and absorbance at wavelengths of 450 and 650 nm was measured using a microplate reader. Percent cytotoxicity was calculated using the following formula: cytotoxicity (%) = [(OD of compound-untreated cells − compound-treated cells)/(OD of compound-untreated cells)] × 100.

### 2.4. Antiviral Effects of Compounds

Confluent fcwf-4 cell monolayers were cultured in medium with or without compounds at the indicated concentrations in 24-well multiplates at 37 °C for 24 h. Cells were washed with MM twice, inoculated with FCoV strain 79-1146 (2000 tissue culture infectious dose (TCID)_50_/0.1 mL per well), and adsorbed at 37 °C for 1 h. After adsorption, cells were washed with MM three times. Cells were cultured in MM without compounds. After cultivation, the culture supernatant was collected and virus titers were measured using the TCID_50_ assay. Cells were collected and stored at −80 °C until RNA isolation as described below.

### 2.5. Time of Addition Assay for PpIX

Confluent fcwf-4 cell monolayers were cultured in 24-well multiplates. Cells were inoculated with FCoV strain 79-1146 (2000 tissue culture infectious dose (TCID)_50_/0.1 mL per well) and adsorbed at 37 °C for 1 h. Following adsorption, the cells were washed three times with MM. The cells were then cultured in MM without compounds for 0–24 h. At designated time points, the culture supernatant was removed, and the cells were washed three times with MM. Subsequently, the cells were cultured in MM containing PpIX (25 μM) until 48 h post-virus inoculation. After cultivation, the culture supernatant was collected. Virus titers and FCoV 3′-UTR levels were measured using the TCID_50_ assay and RT-qPCR, respectively, as described below.

### 2.6. RNA Isolation

Total cellular RNA was extracted from fcwf-4 cells or supernatant using a High Pure RNA Isolation Kit (Roche Diagnostics GmbH, Mannheim, Germany) according to the manufacturer’s instructions. RNA was dissolved in elution buffer.

### 2.7. Quantification of FCoV 3′-UTR

The FCoV gene was quantified by RT-qPCR. RNA was reverse transcribed and amplified using RNA-direct Realtime PCR Master Mix (TOYOBO, Osaka, Japan) with specific primers for FCoV 3′-UTR and GAPDH. Primer and probe sequences are shown in Table 1. The reaction was performed in a total volume of 20 μL/well in 48-well PCR plates using a StepOne Real-Time PCR System (Thermo Fisher Scientific, Waltham, MA, USA) at 90 °C for 30 s, 60 °C for 20 min, and 95 °C for 1 min, followed by 45 cycles at 90 °C for 15 s and 60 °C for 1 min. FCoV 3′-UTR was quantified by absolute quantification and relative quantification. The FCoV 3′-UTR copies were determined using standard FCoV 3′-UTR as described previously [28]. Relative FCoV 3′-UTR was quantified using the comparative Ct method. The Ct value of FCoV 3′-UTR in each sample was normalized with the Ct value of GAPDH (ΔCt). The ΔCt value of each sample was then subtracted from the ΔCt value of the calibrator (ΔΔCt). FCoV 3′-UTR in each sample was calculated as 2^−ΔΔCt^. Statistical analyses were performed with respect to the ΔCt value of each sample.

### 2.8. Measurement of Feline HO-1 Gene Expression

HO-1 gene expression was semi-quantified by RT-PCR. Using total cellular RNA as a template, cDNA was synthesized using ReverTra Ace (TOYOBO Co., Osaka, Japan). Reverse transcription was performed in a final volume of 38 μL containing 1 μL of the oligo (dT) primer (50 μM). The resulting solution was incubated at 42 °C for 30 min to synthesize cDNA. cDNA was amplified by PCR using specific primers for the GAPDH gene and HO-1 gene. Primer sequences are shown in Table 2. PCR was performed in a total volume of 50 μL. One microliter of sample cDNA was mixed with 25 μL of Quick Taq HS DyeMix (TOYOBO Co., Osaka, Japan), 2 μL of 10 μM primer mix, and 22 μL of water. Using a PCR Thermal Cycler Dice (Takara Bio Inc., Shiga, Japan), DNA was amplified at 94 °C for 20 s, followed by 30 cycles of denaturation at 95 °C for 30 s, primer annealing at 60 °C for 45 s, and synthesis at 72 °C for 45 s, with a final extension at 72 °C for 5 min. PCR products were resolved by electrophoresis on 2% agarose gels. Gels were incubated with ethidium bromide solution (1 μg/mL), and bands were visualized using a UV transilluminator at 312 nm and photographed. Band density was quantified under appropriate UV exposure by video densitometry using ImageJ software (version 1.53e, NIH, Bethesda, MD, USA). The HO-1 gene was semi-quantitatively analyzed in terms of the relative density value to that for the housekeeping gene GAPDH.

### 2.9. Direct Antiviral Effects of PpIX

PpIX or DMSO diluted to various concentrations was mixed with FCoV strain 79-1146 (4000 TCID_50_/0.1 mL) at an equivalent volume and reacted at 37 °C for 1 h under light shielding. The virus titer in the mixture was measured using the TCID_50_ assay.

### 2.10. Measurement of Intracellular PpIX Levels

fcwf-4 cells cultured as a monolayer in T-75 flasks were cultured with MM containing a compound for 24 h. Cells were washed in ice-cold PBS 3 times, collected, and stored at −80 °C until the measurement of PpIX levels. Cells were suspended in lysis buffer (20 mM Tris/HCl, 2 mM MgCl_2_, and 1% Triton X-100) containing 125 units/mL of Benzonase Nuclease (Merck KGaA, Darmstadt, Germany) and incubated at 37 °C for 1 h. After the cell suspension was centrifuged at 14,000× *g* at room temperature for 5 min, the supernatant was collected in another tube. The supernatant was mixed with 2.5 μg/mL of Proteinase K (Kanto Chemical Co., Inc., Tokyo, Japan) and reacted at 37 °C for 1 h. The solution was then centrifuged at 14,000× *g* at room temperature for 5 min. An equivalent volume of 10% trichloroacetic acid (Kanto Chemical Co., Inc., Tokyo, Japan) was added to the supernatant obtained, which was placed at room temperature for 10 min. The solution was subsequently centrifuged at 14,000× *g* at room temperature for 5 min. The supernatant was removed and the sediment was collected. Dimethylformamide was added to the sediment obtained and agitated for 10 min. The solution was then centrifuged at 14,000× *g* at room temperature for 5 min. The supernatant was collected, and absorbance at a wavelength of 300 to 800 nm was measured using a BioSpectrometer (Eppendorf, Hamburg, Germany).

### 2.11. Statistical Analysis

Statistical analyses were performed by JMP Pro (version 17.0.0, JMP Statistical Discovery LLC., Cary, NC, USA). Data from only two groups were analyzed using Student’s *t*-test (Welch’s *t*-test) and those of multiple groups by Dunnett’s test. A *p*-value < 0.05 was considered to be significant.

## 3. Results

### 3.1. Cytotoxic Effects of Compounds

The cytotoxic effects of 5-ALA, PpIX, hemin, and SFC on fcwf-4 cells were evaluated using a WST-8 cytotoxicity assay. PpIX, an intermediate metabolite of 5-ALA, has been reported to exert antiviral effects [30]. Ferrous iron is required for the conversion of PpIX to heme. The addition of SFC, a soluble iron source, to 5-ALA has been shown to promote the conversion of PpIX to heme. Briefly, SFC decreases the intracellular level of PpIX, thereby increasing the intracellular level of heme [31]. Heme, a metabolite of PpIX, induces HO-1, an oxygenase known for its antiviral effects [32,33]. Therefore, HO-1 may play a role in the antiviral effects of PpIX.

**Figure 1:**
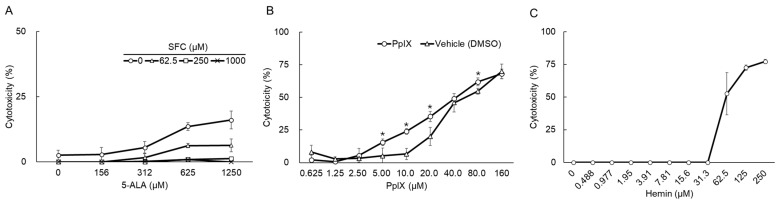
Cytotoxic effects of compounds. fcwf-4 cells were cultured with compounds for 24 h. After the incubation, WST-8 solution was added and cells were incubated for 1 h. The absorbance of formazan produced was measured at 450 nm. Percent cytotoxicity was calculated using the following formula: cytotoxicity (%) = [(OD of compound untreated cells–compound-treated cells)/(OD of compound untreated cells)] × 100. The results obtained are shown as means ± SE. Data represent three independent experiments. (**A**) 5-ALA-SFC combination. (**B**) PpIX *: *p* < 0.05 vs. vehicle. (**C**) Hemin.

The cytotoxic effects of 5-ALA alone and in combination with SFC were examined. The percent cytotoxicity of 5-ALA alone was ≤16.0% (Figure 1). SFC did not exert cytotoxic effects on fcwf-4 cells at any concentration. When fcwf-4 cells were treated with a mixture of SFC and 5-ALA, the cytotoxic effects of 5-ALA decreased in a SFC concentration-dependent manner.

The cytotoxic effects of PpIX increased in a dose-dependent manner (Figure 1B). The 50% cytotoxicity concentration (CC50) of PpIX was 37.2 ± 3.6 μM. In comparisons of PpIX with the vehicle (DMSO), the cytotoxic effects of PpIX in a concentration range of 5 to 20 μM were significantly (≥10%) more potent than those of DMSO.

The cytotoxic effects of hemin at a concentration of ≥31.3 μM increased in a concentration-dependent manner (Figure 1C). The CC50 of hemin was 61.0 ± 8.0 μM.

### 3.2. Antiviral Effects of 5-ALA against FCoV

**Figure 2:**
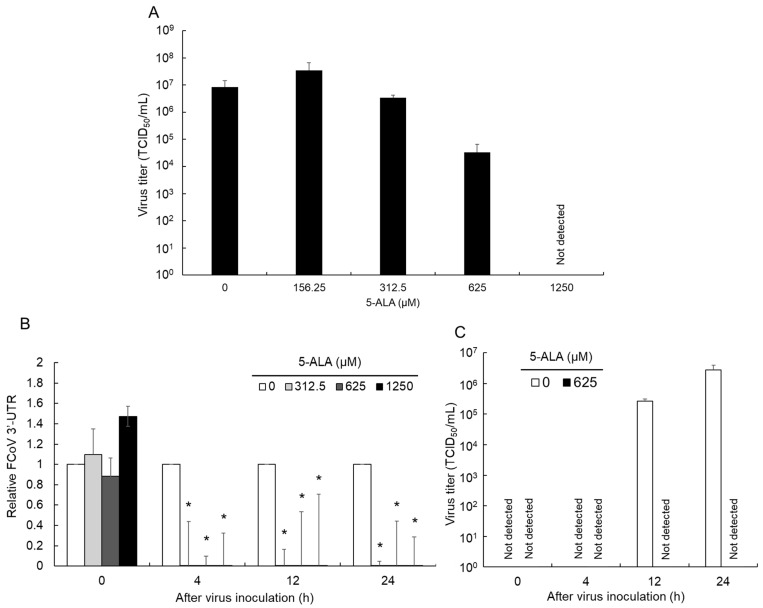
Antiviral effects of 5-ALA. fcwf-4 cells were pretreated with 5-ALA for 24 h. After viral adsorption, cells were cultured in MM without 5-ALA. The results obtained are shown as means ± SE. Data represent three independent experiments. (**A**) The antiviral effects of 5-ALA 48 h after the virus inoculation. (**B**) Relative FCoV RNA replication with different concentrations of 5-ALA. The amount of FCoV RNA was measured using RT-qPCR targeting 3’-UTR and normalized by GAPDH. *: *p* < 0.05 vs. vehicle. (**C**) Virus growth kinetics in fcwf-4 cells pretreated with 5-ALA.

In a previous study, we demonstrated that pre-treating fcwf-4 cells with 5-ALA for 48 h prior to FCoV infection resulted in a significant reduction in virus titer in the culture supernatant [22]. In the current study, we aimed to investigate the antiviral effects of a 24 h pre-treatment with 5-ALA on FCoV infection. Our findings revealed that 5-ALA exhibited antiviral activity in a dose-dependent manner (Figure 2A).

We also examined the inhibitory effects of 5-ALA on the proliferation of FCoV in the initial phase of virus proliferation using FCoV RNA synthesis as an index. No significant difference was observed in FCoV RNA synthesis between 5-ALA-treated and untreated cells 0 h after the FCoV inoculation (Figure 2B). On the other hand, FCoV RNA synthesis was significantly lower in 5-ALA-treated cells ≥ 4 h after the FCoV inoculation than in untreated cells. Similarly, the virus titer in the culture supernatant was determined. The virus titer in the culture supernatant from fcwf-4 cells treated with 625 μM 5-ALA remained below the detection limit for up to 24 h after inoculation (Figure 2C). In contrast, in the absence of 5-ALA, the virus titer began to increase after 12 h.

### 3.3. Antiviral Effects of PpIX against FCoV

**Figure 3:**
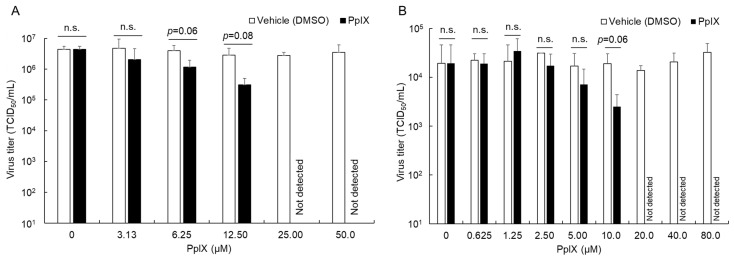
Antiviral effects of PpIX. (**A**) Antiviral effects of PpIX on PpIX-treated cell. fcwf-4 cells were pretreated with PpIX for 24 h. After viral adsorption, cells were cultured in MM without 5-ALA. The virus titer was measured 48 h after the virus inoculation. The results obtained are shown as means ± SE. Data represent three independent experiments. n.s.: no significant. (**B**) Virucidal effects of PpIX against FCoV. PpIX or DMSO was mixed with an equivalent volume of FCoV strain 79-1146 and reacted at 37 °C for 1 h under light shielding. The virus titer in the virus solution was then directly measured using the TCID_50_ assay. The results obtained are shown as means ± SE. Data represent three independent experiments.

We investigated whether PpIX exerts antiviral effects against FIPV. After fcwf-4 cells were pretreated with PpIX for 24 h, FCoV was adsorbed by cells. After 48 h, the virus titer in the culture supernatant was measured. The results obtained showed that it decreased in a PpIX-concentration-dependent manner and was below the detection limit at a PpIX concentration of 25.0 µM or higher (Figure 3A). On the other hand, the virus titer in the culture supernatant from DMSO-treated fcwf-4 cells did not decrease.

PpIX has been shown to exert antiviral effects by interacting with the lipid bilayer membrane of the virus envelope. Therefore, we measured the virus titer of FCoV reacting with PpIX and confirmed whether the virus titer decreased. The results obtained revealed a reduction in a PpIX-concentration-dependent manner (Figure 3B). The virus titer of FCoV that reacted with PpIX at a concentration of 20.0 µM or higher was below the detection limit. On the other hand, the virus titer of FCoV that reacted with DMSO at the same concentrations as those of PpIX solution did not decrease at any concentration.

**Figure 4:**
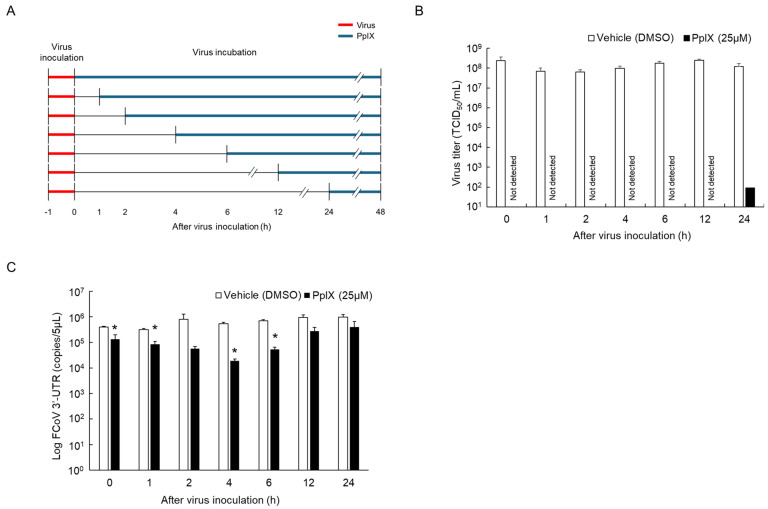
A time of additional assay for PpIX. fcwf-4 cells were infected with the FCoV strain and cultured in medium only. After washing, medium containing PpIX was added at designated times ranging from 0 to 24 h after virus inoculation. Virus titer and FCoV RNA levels were measured 48 h after virus inoculation. (**A**) Schedule for PpIX treatment. (**B**) The virus titer in supernatants. Virus titer was quantified using the TCID_50_ assay. (**C**) The FCoV RNA levels on supernatants. FCoV RNA level was quantified using the RT-qPCR targeting FCoV 3’-UTR. The results obtained are shown as means ± SE. Data represent three independent experiments. *: *p* < 0.05 vs. DMSO.

To confirm the virucidal effect of PpIX, a time-addition assay was conducted (Figure 4A). After FCoV inoculation, PpIX was administered to fcwf-4 cells at various time points, and the virus titer in the culture supernatant was measured 48 h after virus inoculation. The infectious virus in the culture supernatant of fcwf-4 cells treated with PpIX up to 12 h post-inoculation was below the detection limit (Figure 4B). When PpIX was administered 24 h post-inoculation, a slight virus titer (100 TCID_50_/mL) was detected in only one of three independent experiments. In contrast, DMSO treatment resulted in consistently high virus titers of approximately 10^8^ TCID_50_/mL at all time points. Regarding FCoV RNA levels, the culture supernatant from cells treated with PpIX exhibited significantly lower FCoV RNA levels compared to those treated with DMSO up to 6 h after virus inoculation, except for the treatment administered 2 h after virus inoculation (Figure 4C). No significant differences in FCoV RNA levels were observed between PpIX-treated and DMSO-treated cells when PpIX was administered 12 h after virus inoculation or later.

### 3.4. Antiviral Effects of Hemin against FCoV

**Figure 5:**
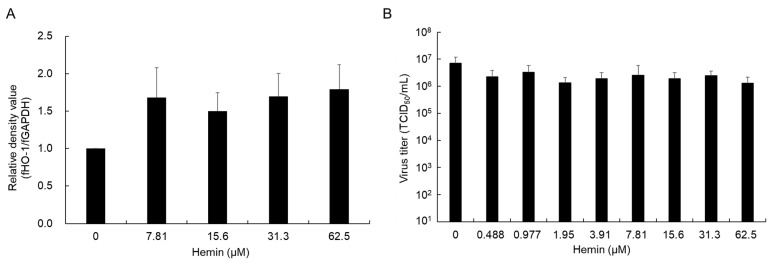
Antiviral effects of hemin. (**A**) The virus titer in cells pretreated with hemin. fcwf-4 cells were pretreated with hemin for 24 h. After viral adsorption, cells were cultured in MM without 5-ALA. The virus titer was measured 48 h after the virus inoculation. (**B**) HO-1 gene expression levels. fcwf-4 cells pretreated with hemin for 24 h. After incubation, cells were collected and HO-1 gene expression levels were semi-quantitatively analyzed relative to GAPDH gene expression. The results obtained are shown as means ± SE. Data represent three independent experiments.

In this study, we investigated whether the induction of HO-1 contributed to antiviral effects against FCoV. To confirm whether HO-1 was induced in fcwf-4 cells treated with the HO-1 inducer, hemin, HO-1 gene expression was semiquantitatively analyzed relative to GAPDH gene expression. In fcwf-4 cells treated with hemin for 24 h, a slight increase was observed in HO-1 gene expression (Figure 5A). The virus titer in the culture supernatant from fcwf-4 cells treated with hemin for 24 h was measured 48 h after FCoV inoculation. No decrease was noted in the virus titer in the culture supernatant regardless of the concentration of hemin (Figure 5B).

### 3.5. Impact of SFC on Antiviral Effects Induced by 5-ALA

**Figure 6:**
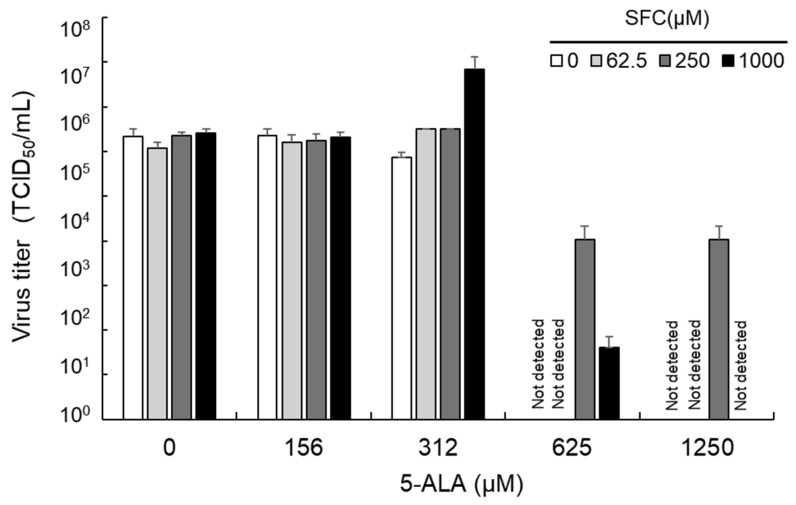
Impact of SFC on antiviral effects of 5-ALA. fcwf-4 cells were pretreated with the 5-ALA-SFC combination for 24 h. After viral adsorption, cells were cultured in MM without 5-ALA and SFC. The virus titer was measured 48 h after the virus inoculation.

We investigated the impact of SFC on the antiviral effects of 5-ALA. fcwf-4 cells were treated with a mixture of 5-ALA and SFC for 24 h. The virus titer in the culture supernatant 48 h after the FCoV inoculation was measured. The virus titer of FCoV in the culture supernatant from fcwf-4 cells treated with 5-ALA alone did not decrease at concentrations of 156 and 312 µM. However, at a concentration of 625 µM or higher, the virus titer was below the detection limit (Figure 6). When 62.5 µM SFC was added with 5-ALA, similar results were obtained. On the other hand, when 250 µM SFC was added with 5-ALA, >10^4^ TCID_50_/mL of the virus was detected even at a 5-ALA concentration of 625 µM or higher. When 1000 µM SFC was added with 5-ALA, no virus was detected at a 5-ALA concentration of 1250 µM; however, 10^3^ TCID_50_/mL of the virus was detected at a 5-ALA concentration of 625 µM.

### 3.6. Effects of Treatment with Each Compound on the Intracellular Level of PpIX

The above results demonstrated the following: (1) 5-ALA exerted antiviral effects against FCoV, (2) an intermediate metabolite of 5-ALA, PpIX, exerted direct antiviral effects against FCoV, (3) hemin induced HO-1, but did not exert any antiviral effect, and (4) SFC, which promotes the metabolism of 5-ALA to heme, reduced the antiviral effects of 5-ALA. Therefore, we hypothesized that the antiviral effects of 5-ALA may be dependent on intracellularly induced PpIX. To verify this, we investigated whether PpIX accumulated intracellularly by treating fcwf-4 cells with each compound.

**Figure 7:**
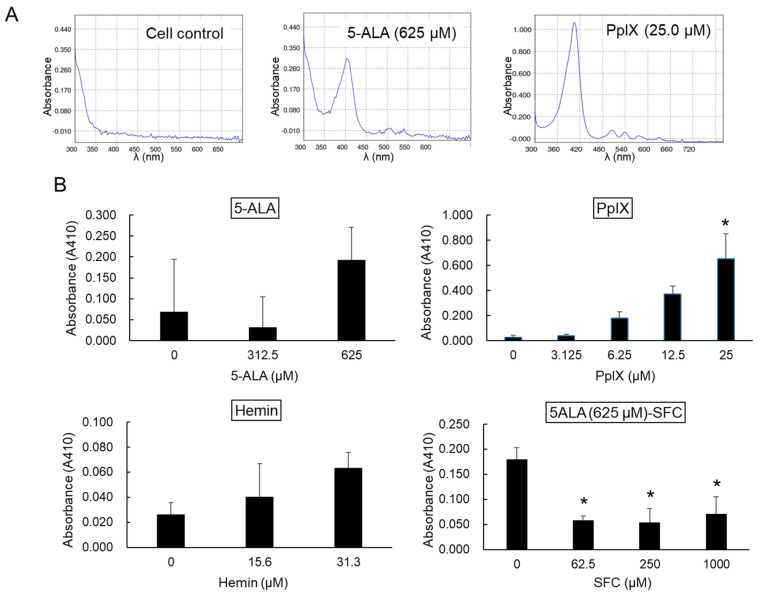
Evaluation of intracellular levels of PpIX in fcwf-4 cells treated with compounds. fcwf-4 cells were cultured with compounds for 24 h. After incubation, cells were collected and lysed in a buffer containing Benzonase Nuclease and incubated at 37 °C for 1 h. The lysate was centrifuged, and the supernatant was treated with Proteinase K at 37 °C for 1 h. After another round of centrifugation, trichloroacetic acid was added to the supernatant, incubated at room temperature for 10 min, and centrifuged again. The resulting sediment was treated with dimethylformamide, agitated, and centrifuged. The final supernatant’s absorbance was measured at 300–800 nm using a BioSpectrometer. (**A**) Absorption spectrum of the supernatant extracted from compound-treated cells. (**B**) Absorbance at 410 nm (A410). Intracellular PpIX levels were quantified by measuring absorbance at 410 nm. The results obtained are shown as means ± SE. Data represent three independent experiments. *: *p* < 0.05 vs. vehicle.

An extract from fcwf-4 cells treated with PpIX for 24 h (fcwf-4 cell extract) showed an absorbance spectrum reaching a peak at a wavelength of approximately 405 nm and several subpeaks at a wavelength ≥ 500 nm (Figure 7A). This was consistent with the previously reported absorbance spectrum of PpIX [34]. Based on the conditions set in our similar experiments conducted using different cells, we quantified the absorbance at a wavelength of 410 nm as the PpIX level. When cells were treated with 625 μM 5-ALA, the same absorbance spectrum was noted. On the other hand, in MM-treated control cells, there was no peak in absorbance at a wavelength of 410 nm. Therefore, the absorbance of an extract from cells treated with each compound at a wavelength of 410 nm was quantified as the concentration of PpIX. There was no increase in absorbance in the cell extract treated with 312.5 μM 5-ALA, whereas that in the cell extract treated with 625 μM 5-ALA increased (Figure 7B). The absorbance of a PpIX-treated cell extract at a wavelength of 410 nm increased in a PpIX concentration-dependent manner. A slight increase was observed in the absorbance of a hemin-treated cell extract. The absorbance of extracts from cells treated with a mixture of 625 μM 5-ALA and SFC at various concentrations was lower than that of an extract from cells treated with 5-ALA alone.

## 4. Discussion

We previously reported that 5-ALA exerted antiviral effects against FCoV; however, the underlying mechanisms remain unclear [22]. 5-ALA generates various metabolites in its intracellular metabolic pathway. A metabolite of 5-ALA, PpIX, intercalates the lipid bilayer membrane, inhibiting interactions between the virus envelope and cell membrane [30]. Briefly, it hinders the process of enveloped virus invasion. PpIX may play an important role in the antiviral effects of 5-ALA. A previous study reported that 5-ALA induced PpIX in cells, exerting PpIX-mediated antiviral effects [25]. In the present study, we showed that PpIX was induced in an established feline cell line at a concentration of 5-ALA at which antiviral effects were observed. In addition, we demonstrated that the proliferation of FCoV was inhibited in fcwf-4 cells following the exogenous application of PpIX and confirmed that PpIX exerted virucidal effects against FCoV. Based on these results, the antiviral effects of 5-ALA against FCoV appear to depend on the intracellular induction of PpIX. Intracellularly accumulated PpIX is excreted extracellularly by ATP binding cassette (ABC) transporters in mitochondrial and cell membranes [35]. Extracellularly released PpIX directly damages virus particles. Since PpIX exerted direct antiviral effects against FCoV, it may have been transported extracellularly, directly inactivating FCoV. However, the present study did not investigate the function or expression of ABC transporters in fcwf-4 cells. Furthermore, the extracellular level of PpIX is unclear. Therefore, we were unable to clarify whether extracellular PpIX exerted antiviral effects. The role of ABC transporters in the antiviral effects of 5-ALA and PpIX against FCoV warrants further study.

In the metabolic pathway of 5-ALA, ferrous iron is required for the conversion of PpIX to heme. SFC is widely used as an iron source of 5-ALA in studies using 5-ALA. When SFC and 5-ALA are simultaneously applied, intracellularly induced PpIX is efficiently converted to heme [31]. In the present study, SFC reduced the intracellular level of PpIX, suppressing the PpIX-dependent antiviral effects of 5-ALA. A previous study reported an SFC-related reduction in antiviral effects against CSFV [25]. In in vivo studies, 5-ALA is often simultaneously administered with SFC. However, based on this result, when SFC is administered at a concentration higher than that of 5-ALA, antiviral effects may not be observed. On the other hand, a low concentration of SFC did not affect the antiviral effects of 5-ALA, but reduced its cytotoxicity. This combination increases the therapeutic index of 5-ALA; therefore, in vivo administration may also be useful. In the future, the antiviral effects of 5-ALA against FCoV need to be investigated in vivo in consideration of the adequate doses of 5-ALA and SFC.

HO-1 decomposes heme converted from PpIX, and induces type I IFN in cells, thereby suppressing virus proliferation [36]. A previous study showed that the HO-1 gene was not induced in 5-ALA-treated fcwf-4 cells; therefore, its role in the antiviral effects of 5-ALA against FCoV was unclear. In the present study, HO-1 gene expression increased in fcwf-4 cells treated with the HO-1 inducer, hemin; however, antiviral effects were not observed. Therefore, the induction of HO-1 may not contribute to antiviral effects against FCoV, at least in fcwf-4 cells. Furthermore, HO-1 decomposes heme, promoting its metabolism to bilirubin, nitric oxide, and iron ions. These metabolites are responsible for immunomodulating and anti-inflammatory activities and exert antiviral effects by preventing apoptosis in virus-infected cells. In other words, the induction of HO-1 may contribute to antiviral effects in vivo [37]. In future studies on the antiviral effects of 5-ALA against FCoV in vivo, the involvement of HO-1 needs to be considered.

In this study, we examined the mechanisms underlying the antiviral effects of 5-ALA against FCoV using compounds involved in the metabolism of 5-ALA and heme biosynthesis. The results obtained suggest that 5-ALA exerted PpIX-dependent, but not HO-1-mediated, antiviral effects against FCoV in fcwf-4 cells. Since the antiviral effects of 5-ALA against FCoV do not target a specific virus protein, the development of a resistant virus may not occur. More detailed investigations on the antiviral effects of 5-ALA and PpIX may be useful for the development of antiviral therapy involving 5-ALA against FCoV.

## Figures and Tables

**Table 1:** Sequences of RT-qPCR primers and probes.

Target	Orientation	Sequence	Reference
Feline GAPDH	For	5′-GCCGTGGAATTTGCCGT-3′	XM_003989222
	Rev	5′-GCCATCAATGACCCCTTCAT-3′	
	Probe	5′6-FAM-CTCAACTAC-ZEN-ATGGTCTACATGTTCCAGTATGATTCCA-3′IABkFQ	
FCoV 3′-UTR	For	5′-GATTTGATTTGGCAATGCTAGATTT-3′	[29]
	Rev	5′-AACAATCACTAGATCCAGACGTTAGCT-3′	
	Probe	FAM-5’-TCCATTGTTGGCTCGTCATAGCGGA -3’BHQ1	

**Table 2:** Sequences of conventional RT-PCR primers.

Target	Orientation	Sequence	Reference
Feline GAPDH	For	5′-GCCGTGGAATTTGCCGT-3′	XM_003989222
	Rev	5′-GCCATCAATGACCCCTTCAT-3′	
Feline HO-1	For	5′-GGTGACCCGGAAAGGATTTA-3′	NM_001009307
	Rev	5′-TTGTTGCGCTCGATCTGT-3′	

## Data Availability

The datasets generated for this study are available on request to the corresponding author.
